# Indirect contacts between Danish pig farms – what are the frequencies and risk-reducing measures, and how can they be used in simulation models?

**DOI:** 10.1186/s13028-024-00789-z

**Published:** 2025-01-24

**Authors:** Mette Fertner, Beate Conrady, Anne Sax Røgind, Elisabeth Okholm Nielsen, Anette Boklund

**Affiliations:** 1 Livestock Innovation, SEGES Innovation P/S, Agro Food Park 15, Aarhus N, DK-8200 Denmark; 2 Department of Veterinary and Animal Sciences, Faculty of Health and Medical Sciences, University of Copenhagen, Grønnegårdsvej 8, Frederiksberg C, DK-1870 Denmark

**Keywords:** Biosecurity, Disease transmission, Pig producers, PRRS, Questionnaire study, SPF system, Transport vehicles, Veterinarians

## Abstract

**Background:**

Information on indirect contacts (e.g. contact with visitors and non-porcine species on farms, shared staff and equipment, contact with trucks) is often poorly recorded even though it constitutes a risk in terms of disease transmission. The aim of the present study was to quantify the number of indirect contacts and associated biosecurity measures in Danish pig herds. A questionnaire survey was conducted among both veterinarians and pig producers in Denmark during 2022–2023. The veterinary questionnaire resulted in 143 answers, representing the Veterinary Health Advisory Service contracts for 53% of non-hobby pig farms. The questionnaire for the pig producers resulted in 373 valid responses and a final response rate of 18%. The results from the veterinary questionnaire provide information on veterinary contacts between pig farms and also estimates on the agreement between registration data and real-life observations.

**Results:**

The questionnaire for veterinarians stated that the majority of veterinarians specialized within pig practice would visit > 3 pig farms per day, with pig farms being located with an average distance between the farms of 15 km. The veterinarians presumed wind, movement of pigs and trucks transporting pigs to be the main routes of PRRS infection. The questionnaire for pig producers provides updated data on indirect contacts (e.g. contact with visitors and non-porcine species on farms, sharing of staff and equipment, procedures for purchase/delivery of pigs and contact with trucks) stratified in terms of farm type and production type. Among respondents, 10% of the pig producers shared staff, while 2% shared equipment (washing robots) with other farms, excluding farms in a joint operation. When purchasing gilts, 70% of the participating pig producers introduced gilts in line with recommendations for strict quarantine for a minimum of 42 days. The delivery of the pigs varied, depending on the type of pigs being delivered: finishers were typically delivered for slaughter through delivery facilities into a (usually empty) slaughterhouse truck, while sows for slaughter were typically delivered by means of a delivery truck offsite into a slaughterhouse truck (usually with other pigs on board).

**Conclusion:**

Since the inclusion of indirect contacts in disease spread models relies on valid data, the present study provided valuable data regarding the frequencies and biosecurity measures of indirect contacts between Danish pig herds, which may be useful in the parametrization of computational epidemiological models.

**Supplementary Information:**

The online version contains supplementary material available at 10.1186/s13028-024-00789-z.

## Background

In the pig industry, biosecurity measures to prevent the introduction of pathogens are considered vital and are well established on many farms [1, 2]. Knowledge on how pathogens enter pig farms and how they spread within the farms is essential for decision makers when implementing mitigating measures on farm, regional or national level. As decision support tools, animal disease spread models can provide useful observations of disease behaviour and capture intricate regionalised spread dynamics. They also allow to investigate the effectiveness of different control measures [3]. Such disease spread models have been developed for endemic diseases (e.g. porcine reproductive and respiratory syndrome (PRRS) [4]) and notifiable contagious diseases (e.g. African swine fever [5], Foot-and-Mouth disease (FMD) [6]). In these models, disease spread is frequently modelled through different pathways related to the specific disease of interest. More specifically, the probability of disease introduction through different pathways is defined by (1) the probability of the infectious pathogen being present in the animal or biological vectors (e.g. mosquitoes, ticks, wildlife) or in/on mechanical vectors (e.g. air, food, equipment), (2) the frequency of the action, such as direct contacts (i.e. the frequency of moving animals on a farm) and/or indirect contacts (i.e. transfer of pathogens mechanically via boots, equipment, rodents), the weather conditions affecting airborne transmission of the pathogen and (3) the mitigating measures taken to reduce the risk of disease introduction (e.g. external biosecurity measures). Between diseases, there will be an overlap in the relevant pathways, although some pathways might be specific for a certain disease.

Previous studies related to the spread of PRRS have identified animal movements, semen, people, equipment, trucks and aerosols [1, 7] as potential sources of transmission between farms. Studies show that the transmission of pathogens by people and equipment can be reduced by implementing biosecurity measures [8]. This is one of the cornerstones in the Danish SPF system. SPF stands for Specific Pathogen Free, and the system is based on the principle that the risk of disease introduction can be reduced through a combination of knowledge on the health status of the herd from which animals are purchased, and high levels of biosecurity. The Danish SPF system is a voluntary system and was launched in 1971.

In 2023, 2,360 Danish farms participated in the ongoing SPF system, representing 48% of Danish non-hobby pig farms (i.e. farms with more than ten sows or with more than 100 pigs in total) [9]. SPF farms monitor for seven pathogens, namely *Mycoplasma hyopneumoniae*, *Actinobacillus pleuropneumoniae*, *Pasteurella multocida*, *Brachyspira hyodysenteriae*, PRRS virus, *Sarcoptes scabiei* var. *suis*, *Haematopinus suis* [10]. Farms may enter different categories (SPF colours) in terms of biosecurity and production type, with nucleus and multiplier farms (referred to as Red SPF farms) having the highest level of biosecurity, and production farms (referred to as Blue SPF or Green SPF) having a “standard” level of biosecurity. A standard level of biosecurity applies to all pig farms with > 300 sows/gilts/boars or > 3,000 finishers or 6,000 weaners (7–30 kg), which need to have a biosecurity plan available [11]. It is the responsibility of the veterinarian holding the Veterinary Health Advisory Service agreement of the farm to go through the biosecurity plan once a year for potential improvements [11]. A set of standards dealing with biosecurity applies to all farms enrolled in the SPF system. Persons entering an SPF farm and SPF livestock trucks must take account of the health status when visiting the farms, there are specified procedures for washing and disinfecting transport vehicles, filters on trucks are required, change of clothes and an entrance lock are required on all farms, the farm buildings must be clearly marked with the declared SPF status including the current disease status, and the entrance door to the farm should be locked. Furthermore, gilts that are introduced are obliged (Red SPF farms) or recommended (Blue SPF farms) to be quarantined for a minimum of 42 days. The purchase of animals from a farm with a low SPF status automatically results in the receiving farm being assigned the same low status. Biosecurity standards are further increased for Red SPF farms in terms of the frequency of testing for diseases, the distance to neighbouring farms and the requirement for a cadaver collection site [10]. Since 2016, all Danish pig farms with a Veterinary Health Advisory Service agreement have been obliged to review on-farm biosecurity procedures annually together with their veterinarian [11]. Furthermore, since 2007 the Danish product standards have ensured legislation and industry requirements for welfare, food safety, traceability in primary production (DANISH Product standard) as well as cleaning and disinfection of trucks from abroad [12].

Traceability of animal movements is required according to EU directive EC/2000/15 [13]. In Denmark, movements of pigs must be recorded in the Central Husbandry Register (CHR) [14]. Therefore, information on direct contact (movement) of potentially infected pigs is already available. In contrast, indirect contacts are usually recorded on farm level in logbooks for visitors, while other contacts, such as trucks and shared equipment, are rarely recorded. Parameters used for the estimation of indirect contacts in current Danish disease spread models are based on old survey data and expert opinions [6, 15, 16]. Farm demographics have changed such that there are now fewer and larger farms. In 2006/2007, approximately 11,500 pig farms were registered, compared with around 8,100 farms in 2018. Furthermore, the median number of sows on a SPF sow farm has been multiplied by four in the same period, from approximately 450 sows to approximately 2,400 sows per farm [17]. Following the changes in farm numbers and sizes, we expect the frequency of indirect contacts to have changed as well. However, the size and direction of such changes might vary with different types of indirect contacts, and have until now been unknown.

The aim of the present study was to quantify the level of indirect contacts and associated biosecurity measures in different types of Danish pig farms (nucleus/multiplier farms with high levels of biosecurity, production farms and organic/hobby farms) using questionnaire surveys among veterinarians and pig producers.

## Methods

The study included two questionnaire surveys: one for Danish veterinarians and one for pig producers (surveys translated from Danish to English are available in the Additional file 4 and Additional file 5). The survey for veterinarians dealt with veterinary contact with and between farms, as well as agreement between registration data and actual observations on-farm. The survey for the farmers dealt with biosecurity in terms of contact with visitors and non-porcine species on farms, staff, equipment, trucks, purchases, and delivery of pigs. Both questionnaires were pre-tested at in-house veterinarians and farmers collaborating on other projects.

### Questionnaire survey – Danish veterinarians

A questionnaire survey was distributed among veterinarians registered on the Danish Pig Industry E-mail list. This list is commonly used to distribute news for veterinarians practicing on pig farms in Denmark. A total of 143 veterinarians were registered on the list and received an online link to the survey in March-April 2022. To increase the response rate, the survey was introduced at meetings in the five largest veterinary pig practices some months prior to the survey. After the survey had been launched, two notifications were distributed in the following weeks.

The questionnaire included the following main subjects: (1) distance travelled by the veterinarians, (2) the level of external biosecurity on Danish pig farms and (3) the response of farm owners to clinical signs of notifiable diseases.

### Questionnaire survey – Danish pig producers

A questionnaire survey was conducted among Danish pig producers and distributed in two ways: firstly, in person at the annual Danish conference for pig producers (*Grisekongressen*) held in October 2022, and secondly in writing via the Danish Pig Industry E-mail list in March-April 2023, which included 2,122 farm owners registered as being DANISH producers in March 2022. Furthermore, the questionnaire survey was mentioned in the Danish magazine for pig producers along with a QR code that provided a direct link to the questionnaire [18]. To increase the response rate, a gift voucher was awarded to one of the participants via a lottery. Hence, name, e-mail address and phone numbers were known on all participants who wanted to participate in the lottery. Some specific questions (e.g. sharing of equipment with other farms) were followed up with an e-mail asking for further details. The questionnaire included the following main subjects: (1) contact with visitors and non-porcine species on farms, (2) shared staff and equipment, (3) contact with trucks, (4) purchase of gilts, and (5) delivery of pigs for transport.

### Data handling

Both questionnaire surveys were created online using the software SurveyExact, whereby the answers were collected instantly. The answers from the two questionnaire surveys were retrieved in two excel files after the collection of responses had been completed on 23 May 2023. Responses stating ‘other’ with a comment in the free text field (e.g. delivery of pigs for sale/slaughter) were, whenever possible, fitted into the pre-defined categories.

Data management and analysis were carried out in R version 4.3.0 [19] using the package readxl [20], dplyr [21] and ggplot2 [22]. A chi-square or Fishers exact test (for counts with less than five in one category) was applied to analyse statistically significant differences between the proportions of questionnaire responses.

## Results

### Responses by Danish veterinarians

A total of 143 veterinarians received the link to the questionnaire survey. Of these, 40 (28%) veterinarians responded, which covered a total of 2,380 Veterinary Health Advisory Service contracts, representing around 53% of all Danish pig farms with more than ten sows or 100 pigs in total (Table 1). All pig farms of a certain size are obliged to enroll in a Veterinary Health Advisory Service contract with a specific veterinarian, meaning that the veterinarian will visit the farm monthly to go through the farm and discuss pig health and welfare, zoonotic aspects and biosecurity measures [11]. Hence, veterinarians specialized within pig practice are expected to hold a number of Veterinary Health Advisory Service contracts. Participating veterinarians held a median of 34 (range 0 to 150) Veterinary Health Advisory Service contracts. Of the participating veterinarians, 34 (85%) practiced strictly on pig farms, while the remaining six (15%) also worked with other animal species. Five of the six largest veterinary pig practices in Denmark were represented.

**Table 1:** Representativeness of pig farmers participating in a questionnaire survey on indirect contacts, spring 2023. The Danish population of pig farms covers all farms registered as active with pigs present in the Central Husbandry Register in September 2023. For each group the absolute numbers (proportions) are given in the table. P-value from chi-square test to test for difference between proportions

		Study population	Danish population of pig farms	*P*-value
Total number of farms		373	6,501	
Farm types	Nucleus/multiplier	17 (0.05)	186 (0.03)	< 0.001
	Production	329 (0.88)	4,177 (0.64)	
	Organic/free-range	13 (0.03)	138 (0.02)	
	Hobby	14 (0.04)	2,000 (0.31)	
Production types^A^	Sow farms	152 (0.41)	1,021 (0.23)	< 0.001
	Integrated farms	38 (0.10)	323 (0.07)	
	Farms with weaners and/or finishers	183 (0.49)	3,157 (0.70)	

^A^ Production types are not defined for hobby farms

**Fig. 1:**
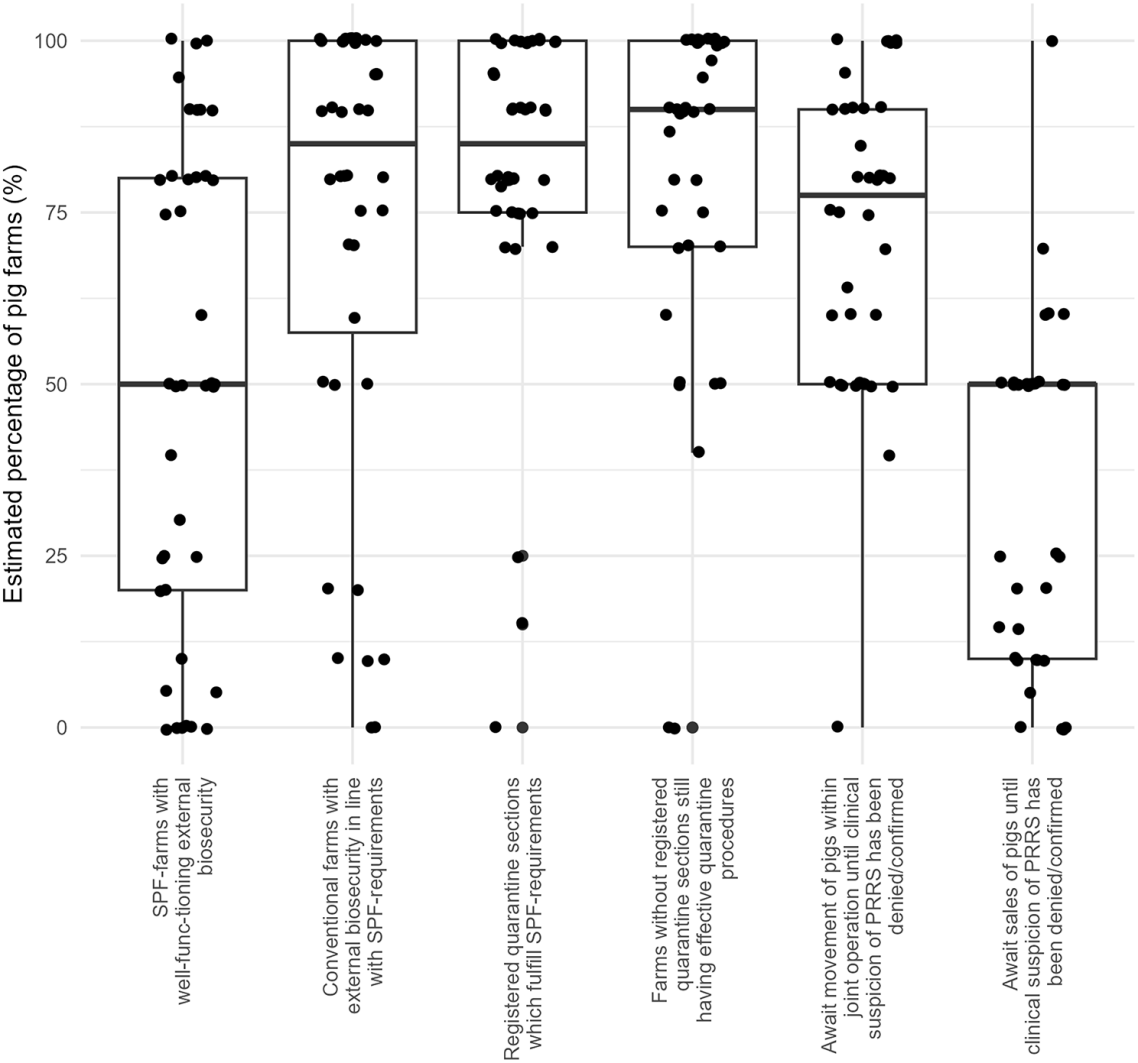
Estimated percentage of pig farms with specific external biosecurity measures. Forty veterinarians have made their best guess on the level of biosecurity in each of the six categories. Every dot represents the guess made by one veterinarian. The y-axis shows the percentage of Danish farms estimated by the veterinarians

**Fig. 2:**
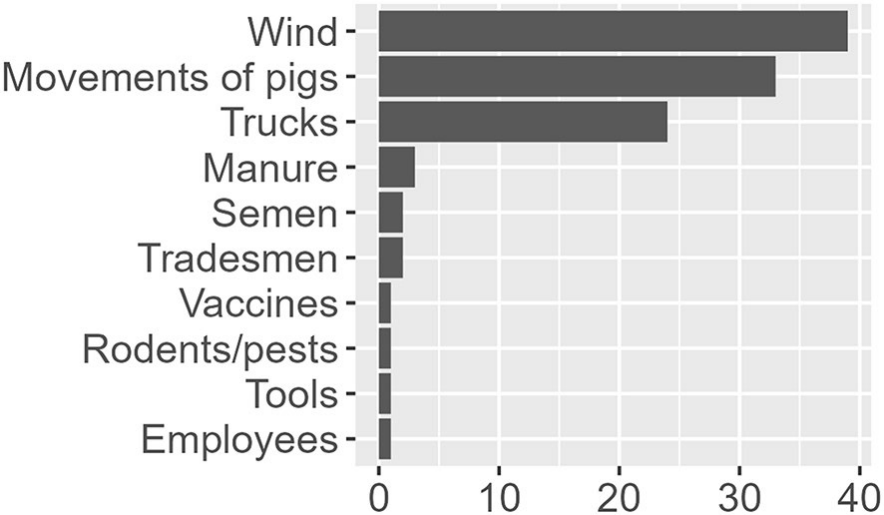
Most likely routes of introduction of PRRS according to 40 Danish veterinarians. Veterinarians were asked to state the three most likely routes of PRRS introduction. Hence, one veterinarian may be represented as one count in up to three categories. The x-axis shows the number of veterinarians reporting the given category

Based on the responses from the participating veterinarians, the average number of daily farm visits included more than three farms/day for 21 (52%) veterinarians, two farms/day for 8 (20%) veterinarians, one farm/day for 5 (13%) veterinarians, and less than one farm/day for 6 (15%) veterinarians. The distance between two visits was 15 km (median; range 0 to 100 km). Thirty (75%) veterinarians stated that they had direct contact with the animals during most of their farm visits. Veterinarians were asked to estimate how many pig farms in their practice followed different external biosecurity procedures (Fig. 1) and which introduction routes they considered most likely for the spread of PRRS (Fig. 2).

### Responses by Danish pig producers

A total of 472 responses from 2,122 e-mails on the receiving list resulted in a response rate of 22%. The following responses were excluded from the analysis: Responses lacking a farm ID (*n* = 14), several responses from the same farm ID with discrepancies between answers (18 farms, including 36 responses), a note that the farm had ceased pig production (*n* = 1) or incomplete responses to more than 75% questions unreported (*n* = 13). To identify inactive farms and retrieve information on the recorded farm size, farm location and SPF farm status, the remaining 408 responses were merged with registration data on active pig farms from the CHR and the SPF register retrieved on 21 September 2023. Inactive farms or farms with no registered pigs (*n* = 35) were subsequently excluded, resulting in a final dataset of 373 responses, giving a final response rate of 18%.

### Representativeness of the study population

Information on the farm size (i.e. number of sows, weaners and finishers), farm type (i.e. nucleus/multiplier farm, production farm, organic/free-range farm) and SPF health status was retrieved from the CHR and SPF registers.

Based on this information, the following farm types were defined:

- Nucleus/multiplier farms: registered as Red SPF farms.
- Hobby farms: with fewer than 10 sows or fewer than 100 pigs in total.
- Organic/free-range farms: as registered in the CHR register, being non-hobby in terms of farm size.
- Production farms: the remaining farms.

In addition, the different production types were distinguished and defined as follows:

- Sow farms with production of either 7–30 kg pigs. Farms with registered sows, where the number of sows equals or exceeds the number of registered finishers. Registered finishers may represent gilts pre-sent on the farm or a minor proportion of pigs that for some reason have not been sold weighing 7–30 kg.
- Integrated farms, with a full-line production of finishers, defined as farms where the number of finishers exceeds the number of registered sows.
- Farms with weaners and/or finishers, i.e. farms with no registered sows, but with registered weaners and/or finishers.

**Fig. 3:**
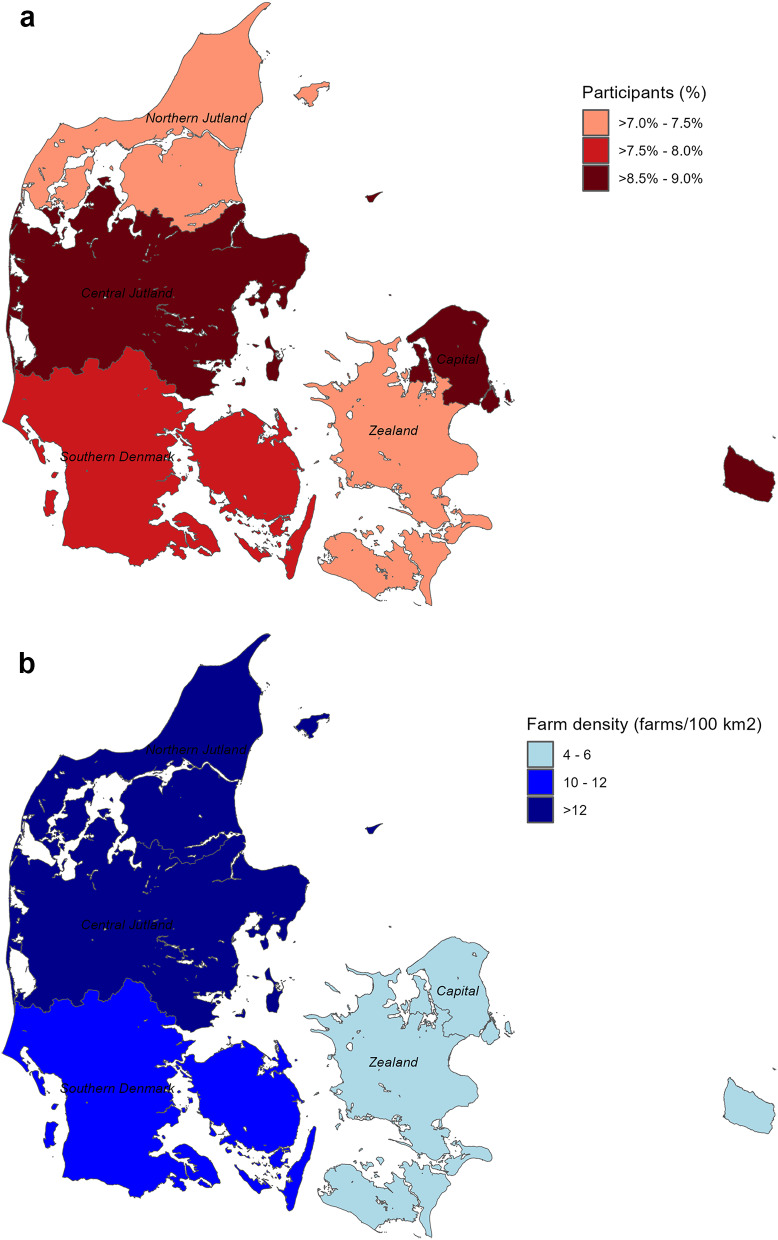
Geographical representativeness of the study population (**A**) and farm density of Danish non-hobby pig farms (**B**). A: The percentage of farms participating in a questionnaire survey in spring 2023 (*n* = 356) among all actively registered pig farms (4,501), with > 10 sows and > 100 pigs in total, stratified in the five Danish regions. B: Farm density (number of farms per 100 km^2^) of all Danish pig farms with > 10 sows and > 100 pigs in total, for each of the five Danish regions. The Capital Region includes the island of Bornholm in the easternmost part of Denmark

The percentage of participating non-hobby farms ranged from 7 to 9% in the five Danish regions (Fig. 3A). The highest percentage of participants was found in Central Jutland and the Capital Region, although large differences in farm density existed between these two regions (Fig. 3B). For the Capital Region, most of the participants originated from the island of Bornholm.

As most responses originated from production farms (Table 1), the result section will analyse this category in more detail and give a brief overview of the remaining farm categories. All detailed results are available in the Additional files (Additional file 1, Additional file 2, Additional file 3).

### Contact with visitors and non-porcine species on farms

Dogs, cats and birds were typically not allowed to enter and exit the housing units on nucleus/multiplier farms, whereas this was allowed on 9% (30/297; two non-responding) of production farms, and 83% (10/12; one non-responding) of organic/free-range farms and 71% (10/14) of hobby farms. Similar findings were observed for the number of visitors: nucleus/multiplier farms recorded a median of two [0;15]_min; max_ visitors per month, production farms recorded a median of one [0;40] _min; max_ visitor per month, organic/free-range farms recorded a median of 4.0 [1;50] _min; max_ visitors per month, and hobby farms a median of 1.5 [0;50] _min; max_ visitors per month. A few of the responding hobby and organic/free-range farms recorded having a substantially high number of visitors (50 visitors per month), since these farms were open to the public. The results, stratified by production type for each of the farm types, are presented in Additional file 1.

### Shared staff and equipment

The sharing of staff and equipment between farms that were not involved in the same joint operation only applied to production farms and one single nucleus/multiplier farm. In total, 10% (38/373) of the participating farms shared staff between the farm of interest and at least one other farm, and a total of 2% (9/373) of farms shared equipment with farms outside the joint operation. Initially, 14 farmers reported sharing equipment with other farms which were subsequently contacted for details on the equipment being shared. Of the 14 farms, nine farms reported washing robots as the equipment being shared, while five farms responded that the answer had been entered incorrectly, as the farm did not share equipment with other farms. Most farmers reported that the washing robot was washed and disinfected between every movement or that the washing robot was shared between farms owned by the same farmer. Of the farmers sharing staff, 29% (11/38) also shared equipment with other farms.

### Trucks for rendering, feed and manure

More farms with sows (sow and integrated farms) had the place of arrival for the rendering truck (cadaver collection point) located > 50 m from the farm compared with farms without sows (farms with weaners and/or finishers). Also, a higher proportion of responding farms with weaners and/or finishers delivered manure for biogas production compared with sow and integrated farms. Although the frequency of delivery seems to be lower for farms with weaners and/or finishers compared with sow farms and integrated farms, feed was usually delivered on a weekly/monthly basis on all farm types (Table 2).

**Table 2:** Contact with trucks among 329 Danish pig farms. Responding pig producers participated in a questionnaire survey carried out in spring 2023 on indirect contacts between Danish pig farms. Farms were categorised as sow farms (*n* = 141), integrated farms (*n* = 25) and farms producing weaners and/or finishers (*n* = 163). Unit m = metres. Absolute numbers (proportions) are presented in the table. P-values from chi-square or fishers exact test (for counts with less than five in one category)

		Sow farms	Integrated farms	Farms with weaners and/or finishers	*P*-value
**Contact with trucks**					
How far from the farm are cadavers collected? (327 responses)	< 50 m	31 (0.22)	6 (0.24)	76 (0.47)	< 0.001
	> 50 m	108 (0.78)	19 (0.76)	87 (0.53)	
How often is feed delivered to the farm? (326 responses)	Daily	3 (0.02)	1 (0.04)	3 (0.02)	0.081
	Weekly	86 (0.62)	16 (0.64)	75 (0.46)	
	Monthly	47 (0.34)	8 (0.32)	81 (0.50)	
	Yearly / Never	3 (0.02)	0 (0.00)	3 (0.02)	
Does the farm deliver manure for biogas? (348 responses)	No	106 (0.76)	19 (0.76)	105 (0.64)	0.065
	Yes	33 (0.24)	6 (0.24)	58 (0.36)	
How often is manure delivered to biogas? (times per month, only for the 97 farms delivering to biogas)	Frequency	8 [0;40]	9 [4;25]	4 [0.5;25]	

### Purchase of gilts

Among all farms, gilts were purchased in 55% (103/186) of farms with registered sows, covering 92 sow farms and 11 integrated farms (Additional file 2). Of these 103 farms, 97 (94%) were production farms (Table 3).

**Table 3:** Quarantine procedures on production farms purchasing gilts. Responses from Danish pig producers participating in a questionnaire survey in spring 2023. Absolute numbers (proportions) are presented in the table. P-values from chi-square or fishers exact test (for counts with less than five in one category)

	Sow farms (*n* = 90)	Integrated (*n* = 7)	*P*-value
Yes, with quarantine of at least six weeks	65 (0.72)	4 (0.57)	0.408
No, inconsistent or less than six weeks of quarantine for purchased gilts^A^	25 (0.28)	3 (0.43)	

^A^ Includes the following procedures for gilt introductions on the farm: no quarantine or not all (majority) gilts being introduced with quarantine of six weeks or more

### Delivery of pigs for transport

Among production farms, 53% (174/329) of the farms sold 7–30 kg pigs to other farms, 64% (209/329) of the farms delivered finishers for slaughter, and 51% (168/329) delivered sows for slaughter.

Of the 174 production farms selling 7–30 kg pigs, 61% (106/174) of the farms delivered these pigs by means of delivery facilities (e.g. delivery room, loading ramp), while 32% (55/174) responded that they used “direct delivery”, i.e. with no delivery facilities. Direct delivery of 7 kg and 30 kg pigs for fattening applied to 7/14 (50%) integrated farms, 43/133 (32%) of the sow herds and 5/20 (20%) of the farms with weaners and finishers (Additional file 3). Three farmers registered direct delivery with a note stating that it was not possible for the pigs to return, while four farmers stated that they used their own truck for transportation, and two farmers stated that the SPF company was responsible for transporting 7–30 kg pigs exiting the farm for fattening elsewhere. Furthermore, these responses relating to direct deliveries of 7–30 kg pigs could also include deliveries for farms within the same joint operation.

Regarding the procedures for collection of finishers for slaughter, five farmers responded that the haulier could enter the housing unit facilities (beyond the delivery area) at the time of collection. Of these five farmers, one was a hobby farmer, while four produced weaners and/or finishers. Of these four farmers, two farmers transported the finishers to the slaughterhouse in their own truck (indicating that the driver is an employee from the farm), while the two other farms producing weaners and/or finishers had their finishers collected by a truck from the slaughterhouse.

**Fig. 4:**
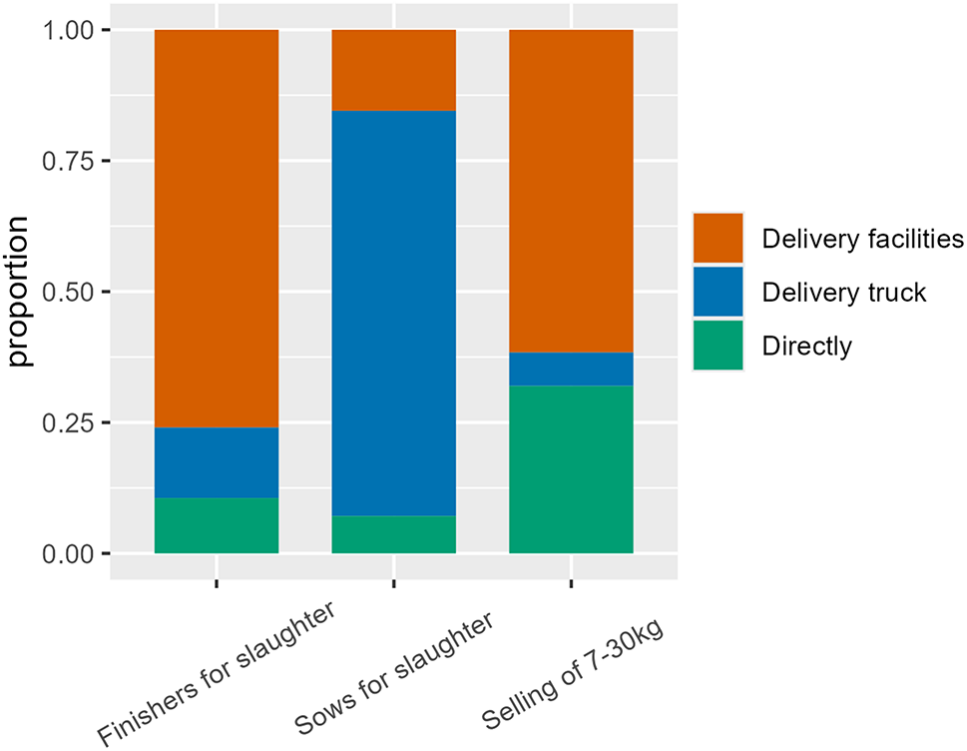
Proportion of farms using a specific delivery method. Delivery methods are presented for pigs for slaughter (finishers and sows) and pigs for fattening (sold at 7–30 kg) among 329 Danish production farms participating in a questionnaire survey in spring 2023. Pigs were delivered from the farm to the truck either directly (green), by means of a delivery truck (blue) or through delivery facilities, e.g. delivery room, loading ramp (orange)

**Fig. 5:**
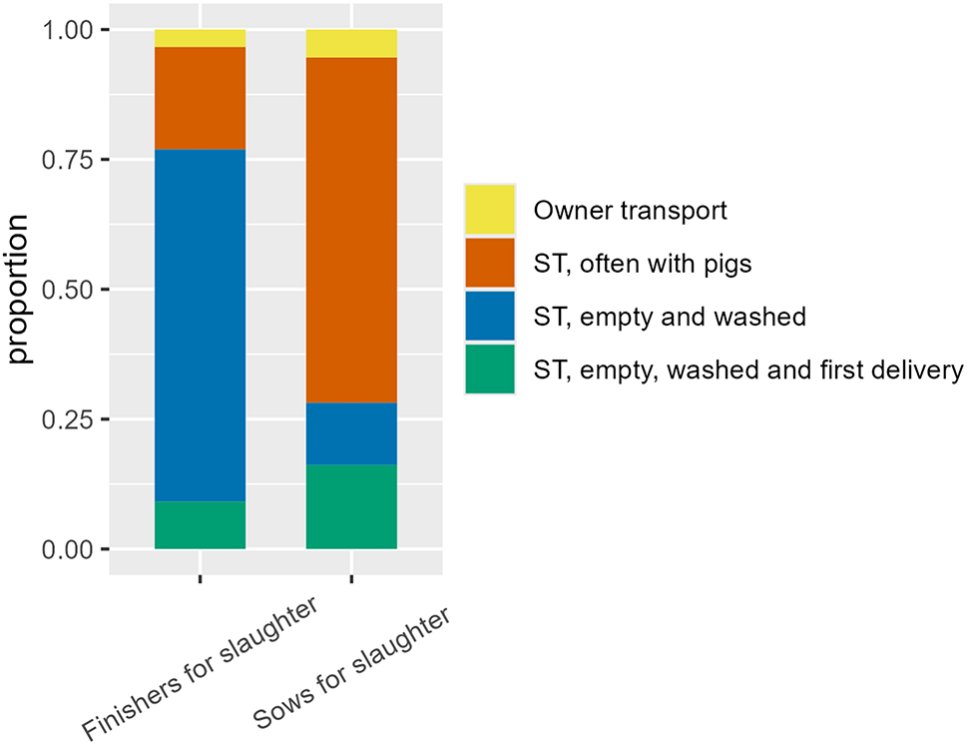
Type of vehicle and requirements for vehicles transporting pigs for slaughter. Results are presented for finishers and sows for slaughter. Proportion of farms given among 329 Danish production farms participating in a questionnaire survey in spring 2023. The requirements for the vehicles include owner transport (yellow), Slaughterhouse truck (ST), often with pigs (orange), Slaughterhouse truck (ST), empty and washed (blue), Slaughterhouse truck (ST), empty, washed and first delivery of the day (green)

Among production farms, sows for slaughter were usually (77%; 114/148) collected from a delivery truck (truck A) (Fig. 4) by a slaughterhouse truck (truck B) with other pigs on board (66%; 111/167) (Fig. 5). A delivery truck is a truck where pigs can be housed for a short period of time while they are waiting to be picked up, e.g. by a slaughterhouse truck. Thereby the slaughterhouse truck does not come into direct contact with the farm. Typically, the delivery truck belongs to the farmer himself. Only few farmers were responsible for transporting their sows and finishers for slaughter (5% (9/167) and 3% (7/208), respectively) (Fig. 5). In those cases where the owner transported sows for slaughter from production farms most of the owners (8/9) transported the sows from the farm to a delivery truck, thereby avoiding direct contact with the slaughterhouse facilities. However, only 14% (1/7) of finishers for slaughter transported by the owner were transported by means of a delivery truck, which means that the remaining deliveries (86% (6/7)) might have included contact between the farmer’s truck and the slaughterhouse facilities.

**Fig. 6:**
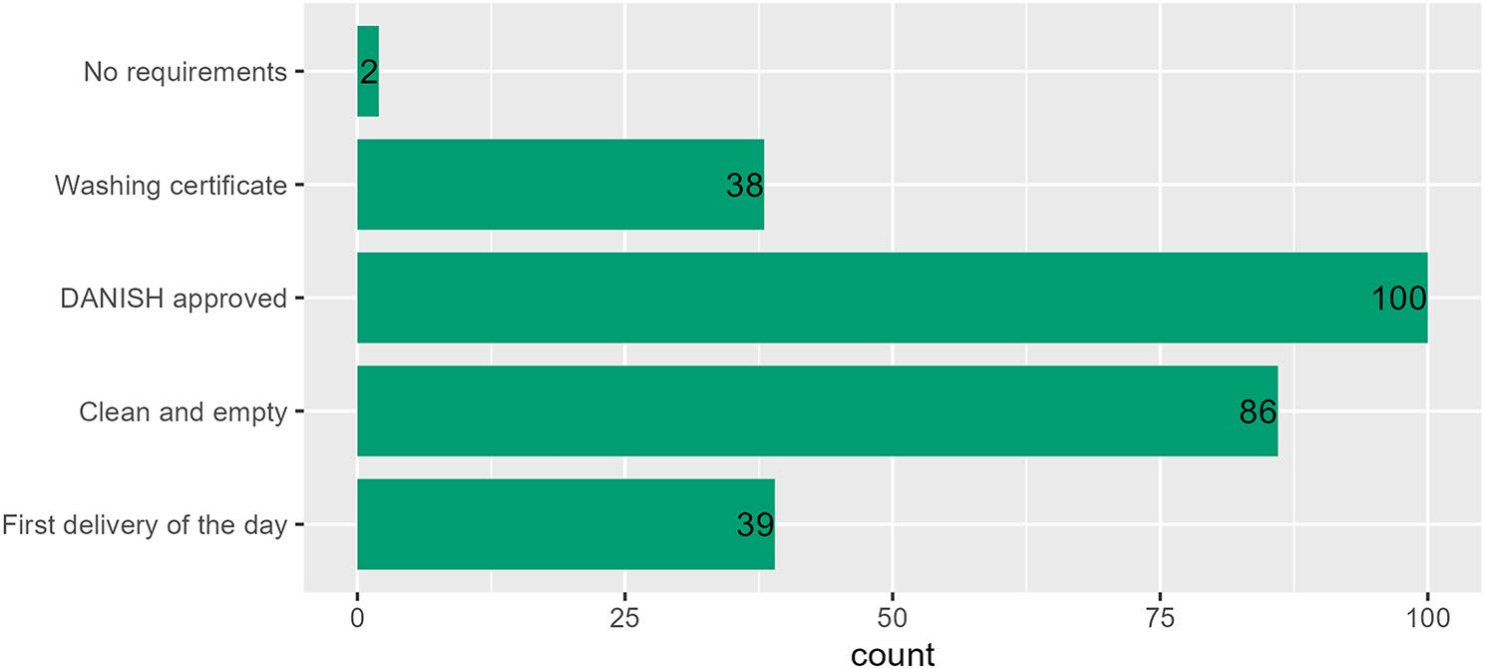
Requirements for vehicles arriving at the pig farm and transporting pigs for fattening (selling 7–30 kg). Responses by 265 farmers among 329 Danish production farms participating in a questionnaire survey in spring 2023

Most of the respondents stated that they had requirements for the trucks arriving at their farm. Most of the farmers required the truck to be DANISH approved, meaning that the hygiene and check of washing certificates procedures had been followed [12]. Two production farms responded that they had no requirements for the vehicles on arrival (Fig. 6) and stated that the transporting company (SPF company and ‘other pig transporter’) was responsible for the transport of pigs exiting the farm.

## Discussion

With the present paper, we aimed to characterise the level of indirect contacts and associated biosecurity measures among pig farms through questionnaire surveys targeting veterinarians specializing in pigs and pig producers.

A participation rate of 28% was obtained in the questionnaire survey targeting Danish pig veterinarians. The link used for the distribution of the survey also included veterinarians who were not relevant to the survey, such as veterinarians not specializing in pigs. Therefore, the participation rate among Danish pig veterinarians seems underestimated. This is highlighted by the fact that the 40 participating veterinarians represented more than half of the Health Advisory Service contracts for Danish pig farms, mainly representing industrialized pig production. A higher participation rate as well as a high representation of pig farms would most likely reduce the uncertainty related to answers from veterinarians. As for the questionnaire aimed at the pig producers, the total number of participants represented 18% of the farmers on the e-mail list and 6% (373/6,501) of all Danish pig farms currently active in the CHR register. Production farms and nucleus/multiplier farms were well represented among the participating farms, as opposed to hobby farms, possibly due to the distribution of the questionnaire survey through channels targeting commercial pig production (DANISH registered farms). Furthermore, sow farms were overrepresented, which might be explained by their particular interest in biosecurity and the risk of disease being introduced on these farms, based on the more severe consequences related to disease.

In general, the survey may have been biased, since the producers might have known how to “answer correctly” and therefore did not represent the actual on-farm procedures. Incongruence between answers and actual practices have been identified in other studies previously [23]. Alternatively, we could have made an “on-farm” questionnaire, which might have resulted in a higher response rate but substantially fewer responses in total. Biosecurity and management are ongoing procedures and are repeated on a daily basis. On-farm questionnaires or even an inspection of farm procedures would only give a snapshot of the farm routines, and therefore no type of survey is perfect for investigating biosecurity and farm management. Implementing biosecurity and good farm management takes a daily effort, taking time to learn, and over time there is a risk of a gradual decrease in standards. Therefore, frequent control and inspiration are important to maintaining a generally high standard.

The aim of the present study was to quantify the number of indirect contacts and associated biosecurity measures. Hence, the risk of disease transmission by each contact, depends on the level of preventive measures attached to it. The veterinarians expressed some variation in answers (Fig. 1), although a previous study found that Danish pig farms generally had a higher level of external biosecurity and lower between-farm variation compared with other countries (Belgium, France, Germany, the Netherlands, and Sweden) [24]. Historically, the Danish pig industry has focused on biosecurity due to the SPF system, which was launched in the 1970s. This may explain why the majority of veterinarians found a high percentage of conventional pig farms to have external biosecurity in line with SPF-requirements (Fig. 2).

Registration data, such as farm demographics and animal movements, are widely used in disease spread models, since they typically cover a large proportion of the population of interest. However, discrepancies between registration data and real-life observations are an ongoing concern [25]. Therefore, the estimations made by veterinarians regarding these discrepancies are extremely important, since veterinarians have a general overview of several pig farms, yet answers may be affected by the subjective judgement of the veterinarian. In general, the responding veterinarians found a high agreement between SPF status and effective external biosecurity and, to some degree, also conventional pig farms. Likewise, veterinarians found a high agreement between the absence/presence of a registered quarantine unit and the actual quarantine procedures (Fig. 1). In addition, the veterinarians provided input on which parameters they considered important in terms of developing a disease spread model for PRRS (Fig. 2). This is important information and could be used to generate future research questions and to prioritize pathways in simulation models. However, the answers cannot stand alone. Participating veterinarians may be affected by information newly presented as relevant in national magazines and may miss fundamental knowledge on the specific disease transmission which should also be handled in the simulation model.

Data regarding indirect contacts are often not recorded or available at all, although they are essential in weighting the different transmission pathways in disease spread models and helping to identify effective mitigation measures against the disease spread. Information on biosecurity measures and the frequency of their use is important in terms of scaling the importance of these indirect contacts. In the present study, we have described indirect contacts and the related biosecurity measures regarding (1) contact with visitors and non-porcine species on farms, (2) shared staff and equipment, (3) contact with trucks, (4) purchase of gilts, and (5) delivery of pigs for transport, and we will discuss the results and their applications separately in the following sections.

### Contact with visitors and non-porcine species on farms

Contact with visitors and non-porcine species on farms, included dogs/cats and birds that are allowed to enter and exit the farms. Dogs, cats and birds were generally only allowed to enter a minority of the responding production farms (4–10%). On these farms, these species are allowed to enter possibly because the facilities are old and experience nonoptimal compliance with biosecurity measures. However, none of the responding nucleus/multiplier farms allowed dogs, cats or birds to enter the farm. Nucleus/multiplier farms have more restrictive biosecurity measures and may also expect more severe consequences in the case of disease outbreaks. A large part of the responding organic/free-range farms allowed dogs, cats, and birds to enter, probably because of the housing unit design and the pig’s freedom to go outside, thereby making it difficult to prevent “uninvited visitors” such as dogs, cats and birds from entering the facility.

Similarly, the number of visitors during a typical month of production and nucleus/multiplier farms were lower compared with organic/free-range farms, probably due to the generally higher level of biosecurity and the risk of transmission of infectious diseases. For several diseases, humans have been described as a mechanical vector or a risk factor [26–29], carrying pathogens on clothes, boots, skin or even in nostrils [9, 30], thereby increasing the risk for the visited farms. A few of the production and organic/free-range farms were “open-to-visitors farms”, which explains the high number of visitors to these farms. An open farm strategy may challenge restrictive biosecurity measures, and this can lead to an increased risk of the introduction of disease.

### Shared staff and equipment

Approximately half of the responding production farms were enrolled in a joint operation. According to SPF rules, farms in a joint operation are allowed to share staff and equipment with one another, since these farms are considered one unit of production and therefore always share the same health status [31]. A few farms responded that they share staff and/or equipment with other farms not enrolled in a joint operation. However, these were often farms with the same owner. Based on the results from the present study, one may speculate whether there is a higher risk of disease transmission between farms of the same ownership.

### Trucks for rendering, feed, and manure

Production farms with weaners and/or finishers seemed to have a shorter distance between the farm and the collection point for the cadavers (< 50 m), which means that the rendering truck would be in closer proximity to the farm. For red SPF farms, the cadaver collection point should be situated as far as possible from the farm, though with a minimum distance of 50 m. Blue SPF farms should have the cadaver collection point situated as far as possible from the farm without any specified distance [31]. Since the rendering truck does not take the health status into account when visiting the farm, having the collection point located in near proximity to the farm may constitute a risk. The risk related to rendering trucks has been described for several diseases. Rendering trucks and trucks transporting pigs to the slaughterhouse have previously been associated with PRRS infections in Hungary [32], and on poultry farms the use of rendering or offsite disposal of cadavers has been described as a risk factor related to the highly pathogenic avian influenza [33]. In Denmark, it is common to have a cooling facility or a specially designed collection point for cadavers located far from the housing units, as described in the results, and this should reduce the risk from the trucks. However, cooling facilities and collection points are sometimes shared between several farms, either with shared ownership or joint operations, resulting in frequent contacts between each farm and the collection point. Handling of cadavers should be carried out as the final task of the day to avoid cross-contamination. However, risky movements of farm staff, e.g. movement of staff from the cadaver storage to live pigs inside the stable have previously been studied in five farrow-to-finish farms [23]. Here, risky movements were found to occur in all farms with varying frequency (from 9 to 38%).

The delivery of manure for biogas has increased substantially during the past decade, and this trend is expected to continue [34]. There is concern as to whether trucks transporting manure for biogas constitute a risk in terms of disease transmission, since the health status is not taken into account during the herd visits. In particular, the risk of infected return air when loading the truck with manure has been a subject of some concern in terms of airborne pathogens such as PRRS [35]. To the best knowledge of the authors, this is the first paper to describe the collection frequencies and distribution of pig herds currently enrolled in the delivery of manure for biogas.

### Purchase of gilts

Quarantining purchased gilts in line with recommendations, whereby all gilts entering the farm are kept in a separate quarantine unit for a minimum of 42 days, was performed for 72% (65/90) of sow farms and 57% (4/7) of integrated farms (Table 3). To be effective, quarantining needs to be consistent and the facilities situated far enough from the main housing unit [31]. Furthermore, in order to ensure that purchased animals are not infected, the time spent in quarantine needs to be sufficiently long for the animals either to show clinical signs or to be tested negative during the quarantine period or tested negative on the farm of origin. The SPF recommendations of 42 days of quarantine are somewhat longer than the recommendations in e.g. Belgium of 28 days [36]. Since purchased gilts are a direct route of transmission if they are not thoroughly quarantined, this introduction pathway constitutes a greater risk related to the individual event, compared with indirect transmission routes. Therefore, the use of quarantine is of utmost importance.

### Delivery of pigs for transport

The delivery of pigs for transport may be a complex procedure to narrow down to just a few questionnaire categories. As for any area of biosecurity, it is ultimately the responsibility of the staff to diligently adhere to the recommendations, where the SPF-biosecurity recommendations seem to set the standards of biosecurity among Danish pig farms although not all farmers are enrolled in the system. Specifically, when loading pigs, they must ensure that there is no direct or indirect contact between the truck and the farm. The level at which the biosecurity measures are followed has a considerable impact on the risk associated with the delivery of pigs or with any other procedure on the farm. Also, it would have been beneficial to combine the answers with management procedures, e.g. whether all-in/all-out procedures were carried out for farms where the haulier was allowed to enter the housing unit facilities.

In the present study, delivery was categorized as “Direct”, “Delivery facilities” and “Delivery truck”. Direct delivery of pigs may be associated with a risk of pigs returning to the housing unit area, unlike with the use of delivery facilities. However, several of the farm owners specifically responded that the return of pigs to the housing unit was not possible, despite the use of direct delivery of pigs. On the contrary, delivery facilities are no better than the employees using them. For example, if doors between delivery facilities and housing units are not closed properly, or separation zones are not respected, the risk is not reduced to the level we would normally expect. While “Direct delivery” and “Delivery facilities” are present on-farm, the “Delivery van” is driven to an area outside or on the edge of the farm area for the arrival of the slaughterhouse truck. The use of a “Delivery van” is associated with additional work and is typically used for the delivery of a relatively small number of pigs, i.e. a number of pigs that cannot fill up an entire truck. Therefore, one could expect the truck to arrive at the delivery van already carrying pigs from other farms, which was also reported in this survey. Among production farms responding to the survey, sows were often delivered for slaughter with other pigs on board the slaughterhouse truck, which may be of minimal risk due to the concurrent widespread use of delivery trucks. A few farms with weaners and/or finishers also responded regarding the delivery of sows for slaughter (one nucleus/multiplier farm and five production farms), which indicates an inconsistency between the registration data and the actual presence of pigs on the farm. However, it is not unusual for farms with weaners and finishers to keep a few sows.

## Conclusions

In this study, we have collected important information about the frequency of contacts and biosecurity on Danish pig farms to be used in risk assessments and disease spread models, and we have also reinforced the focus on biosecurity among Danish pig farmers and veterinarians. Despite a general description of delivery methods, and biosecurity requirements on-farm, the results elucidated a tendency towards a higher degree of indirect contacts, such as staff and equipment, between farms of the same ownership. Also, despite the recommendations, 28% of the sow farms diverged from consistent use of quarantine in the insertion of gilts.

## Electronic supplementary material

Below is the link to the electronic supplementary material.

Additional file 1. Indirect contacts between Danish pig farms in terms of staff, visitors, shared equipment, and trucks. Results from a questionnaire survey among 373 Danish pig producers carried out in spring 2023. The unit m represents metres. Numbers (proportions) are presented in the table.

Additional file 2. Farms purchasing gilts and responsible for transporting unit for purchased pigs. Results are presented for Danish sow and integrated pig farms participating in a questionnaire survey in spring 2023. Numbers (proportions) are presented in the table.

Additional file 3. Delivery of pigs for fattening and slaughter on 373 Danish pig farms. The mentioned farms participated in a questionnaire survey in spring 2023. Numbers (proportions) are presented in the table.

Additional file 4. Questionnaire for veterinarians (translated from Danish)

Additional file 5. Questionnaire for pig farmers (translated from Danish)

## Data Availability

The datasets generated and analysed in the current study are not publicly available due to the protection of privacy of the study participants.

## Declarations

### Prior publication

Data have not been published previously.
