# Supplementary material for: Indirect contacts between Danish pig farms – what are the frequencies and risk-reducing measures, and how can they be used in simulation models?
Source: Acta Vet Scand. 2025 Jan 24;67:7. doi: 10.1186/s13028-024-00789-z (PMC11762108; doi:10.1186/s13028-024-00789-z)
Supplement: Supplementary file 1 — Additional file 1. Indirect contacts between Danish pig farms in terms of staff, visitors, shared equipment, and trucks. Results from a questionnaire survey among 373 Danish pig producers carried out in spring 2023. The unit m represents metres. Numbers (proportions) are presented in the table. [file 13028_2024_789_MOESM1_ESM.docx]

**Additional file 1. Indirect contacts between Danish pig farms in terms of staff, visitors, shared equipment and trucks.** Results from a questionnaire survey among 373 Danish pig producers carried out in spring 2023. The unit m represents metres. Numbers (proportions) are presented in the table.

|  |  |  | Nucleus/multiplier (n= 17) | | | Production farms (n=329) | | | Organic/free-range (n=13) | | | Hobby (n=14) | Total responses |
| --- | --- | --- | --- | --- | --- | --- | --- | --- | --- | --- | --- | --- | --- |
|  |  |  | Sow farms | Integrated | Weaners and/or finishers | Sow farms | Integrated | Weaners and/or finishers | Sow farms | Integrated | Weaners and/or finishers | NA |  |
|  |  |  | *4* | *8* | *5* | *141* | *25* | *163* | *4* | *4* | *5* | *14* | *373* |
| Total number of pigs ^B^ |  |  | 2,303  [550; 3,720] | 3,443  [1,740; 8,600] | 1,500  [1,200; 3,350] | 2,760  [415; 16,150] | 3,125  [1,207; 8,955] | 2,000  [300; 13,750] | 2,278  [2,050; 5,000] | 2,380  [481; 3,100] | 1,100  [270; 2,100] | 4  [1;] | 373 |
| **Visitors** |  |  |  |  |  |  |  |  |  |  |  |  |  |
| Dogs/cats allowed to enter and exit the housing unit (N=373) | Yes |  | 0 (0.00) | 0  (0.00) | 0  (0.00) | 12  (0.09) | 2 (0.08) | 16  (0.10) | 4  1.00) | 4 (1.00) | 2  (0.40) | 10  (0.71) | 50 |
|  | No |  | 4  (1.00) | 8  (1.00) | 5  (1.00) | 128  (0.91) | 23 (0.92) | 146  (0.90) | 0 (0.00) | 0 (0.00) | 2  (0.40) | 4  (0.29) | 320 |
| Birds allowed to enter and exit the housing unit (370 responses) | Yes |  | 0 (0.00) | 0  (0.00) | 0 (0.00) | 11 (0.08) | 1 (0.04) | 10 (0.06) | 4 (1.00) | 4 (1.00) | 5 (1.00) | 11 (0.79) | 46 |
|  | No |  | 4 (1.00) | 8 (1.00) | 5 (1.00) | 127 (0.92) | 24 (0.96) | 153 (0.94) | 0 (0.00) | 0 (0.00) | 0 (0.00) | 3 (0.21) | 324 |
| Visitors (373 responses) | Number responses |  | 4 | 8 | 5 | 141 | 25 | 163 | 4 | 4 | 5 | 14 | 373 |
| Number of visitors^A^ (consultants, tradesmen) entering the housing unit during a typical month ^B^ |  |  | 1 [1;3] | 3.5  [1;15] | 2  [0;6] | 2  [0;10] | 1  [0;7] | 1  [0;40] | 6 [3;50] | 5 [3;7] | 3 [1;50] | 1.5 [0;50] |  |
| **Sharing of staff and equipment** |  |  |  |  |  |  |  |  |  |  |  |  |  |
| Employees working on other farms (373 responses) | Yes |  | 0 (0.00) | 0 (0.00) | 1 (0.20) | 17 (0.12) | 2 (0.08) | 18 (0.11) | 0 (0.00) | 0 (0.00) | 0 (0.00) | 0 (0.00) | 38 |
|  |  | Farms enrolled ^B^ | - | - | 2 [2;2] | 3  [1;5] | 3  [2;4] | 3 [1;5] | - | - | - | - |  |
|  |  | Distance ^B^ | - | - | NA | 3  [0.4;10] | 5.5  [4;7] | 10 [0;20] | - | - | - | - |  |
|  | Yes, farms in joint operation |  | 3 (0.75) | 4 (0.50) | 2 (0.40) | 68 (0.48) | 11 (0.44) | 77 (0.47) | 2 (0.50) | 3 (0.75) | 0 (0.00) | 1  (0.07) | 171 |
|  | No |  | 1 (0.25) | 4 (0.50) | 2 (0.40) | 56 (0.40) | 12 (0.48) | 68 (0.41) | 2 (0.50) | 1 (0.25) | 5 (1.00) | 13  (0.93) | 164 |
| Sharing of equipment with other farms (373 responses) | Yes |  | 0 (0.00) | 0 (0.00) | 0 (0.00) | 3 (0.02) | 1 (0.04) | 10 (0.06) | 0 (0.00) | 0 (0.00) | 0 (0.00) | 0 (0.00) | 14 |
|  |  | Farms enrolled ^B^ | - | - | - | 4 [3;5] | 2  [2;2] | 3 [2;4] | - | - | - | - |  |
|  |  | Distance between farms enrolled ^B^ | - | - | - | 3  [1;7] | 7  [7;7] | 10 [0.5;10] | - | - | - | - |  |
|  | Yes, farms in joint operations |  | 1 (0.25) | 0 (0.00) | 0 (0.00) | 25 (0.18) | 3 (0.12) | 32 (0.20) | 0 (0.00) | 2 (0.50) | 1 (0.20) | 0 (0.00) | 64 |
|  | No |  | 3 (0.75) | 8 (1.00) | 5 (1.00) | 113 (0.80) | 21 (0.84) | 121 (0.74) | 4 (1.00) | 2 (0.50) | 4 (0.80) | 14 (1.00) | 295 |
| **Contact with trucks** |  |  |  |  |  |  |  |  |  |  |  |  |  |
| How far from the farm are carcasses collected? (371 responses) | < 50 m |  | 1  (0.25) | 0 (0.00) | 1 (0.20) | 31 (0.22) | 6 (0.24) | 76 (0.47) | 0 (0.00) | 1 (0.25) | 1 (0.20) | 3 (0.21) | 120 |
|  | > 50 m |  | 3  (0.75) | 8 (1.00) | 4 (0.80) | 108 (0.78) | 19 (0.76) | 87 (0.53) | 4 (1.00) | 3 (0.75) | 4 (0.80) | 11 (0.79) | 251 |
| How often is feed delivered to the farm? (370 responses) | Daily |  | 1  (0.25) | 0  (0.00) | 0  (0.00) | 3  (0.02) | 1  (0.04) | 3  (0.02) | 0  (0.00) | 1  (0.25) | 0  (0.00) | 1  (0.07) | 10 |
|  | Weekly |  | 3  (0.75) | 6  (0.75) | 4  (0.80) | 86  (0.62) | 16  (0.64) | 75  (0.46) | 3  (0.75) | 2  (0.50) | 3  (0.60) | 1  (0.07) | 199 |
|  | Monthly |  | 0  (0.00) | 2  (0.25) | 1  (0.20) | 47  (0.34) | 8  (0.32) | 81  (0.50) | 1  (0.25) | 1  (0.25) | 2  (0.40) | 5  (0.36) | 148 |
|  | Yearly |  | 0  (0.00) | 0  (0.00) | 0  (0.00) | 1  (0.01) | 0  (0.00) | 0  (0.00) | 0  (0.00) | 0  (0.00) | 0  (0.00) | 3  (0.21) | 4 |
|  | Never |  | 0  (0.00) | 0  (0.00) | 0  (0.00) | 2  (0.01) | 0  (0.00) | 3  (0.02) | 0  (0.00) | 0  (0.00) | 0  (0.00) | 4 (0.29) | 9 |
| Does the farm deliver manure for biogas? (369 responses) | Yes |  | 1 (0.25) | 3  (0.38) | 2 (0.40) | 33 (0.24) | 6 (0.24) | 58 (0.36) | 1 (0.25) | 1 (0.25) | 0 (0.00) | 0 (0.00) | 105 |
|  | How often is manure delivered to biogas? (times per month, only for the 105 farms delivering to biogas) | Frequency | 2  [2;2] | 9  [5;10] | 4 [4;4] | 8 [0;40] | 9 [4;25] | 4  [0.5;25] | 10  [10;10] | 4  [4;4] | - | - |  |
|  | No |  | 3 (0.75) | 5  (0.63) | 3 (0.60) | 106 (0.76) | 19 (0.76) | 105 (0.64) | 3 (0.75) | 3 (0.75) | 5 (1.00) | 12 (1.00) | 264 |
