# Supplementary material for: Indirect contacts between Danish pig farms – what are the frequencies and risk-reducing measures, and how can they be used in simulation models?
Source: Acta Vet Scand. 2025 Jan 24;67:7. doi: 10.1186/s13028-024-00789-z (PMC11762108; doi:10.1186/s13028-024-00789-z)
Supplement: Supplementary file 2 — Additional file 2. Farms purchasing gilts and responsible for transporting unit for purchased pigs. Results are presented for Danish sow and integrated pig farms participating in a questionnaire survey in spring 2023. Numbers (proportions) are presented in the table. [file 13028_2024_789_MOESM2_ESM.docx]

**Additional file 3. Delivery of pigs for fattening and slaughter on 373 Danish pig farms participating in a questionnaire survey** in spring 2023. Numbers (proportions) are presented in the table.

|  |  |  | Nucleus/multiplier (n=17) | | | Production farms (n=329) | | | Organic/free-range (n=13) | | | Hobby (n=14) | Total responses |
| --- | --- | --- | --- | --- | --- | --- | --- | --- | --- | --- | --- | --- | --- |
| **Pigs for fattening** |  |  | Sow farms | Integrated | Weaners and/or finishers | Sow farms | Integrated | Weaners and/or finishers | Sow farms | Integrated | Weaners and/or finishers | NA |  |
| Farms delivering pigs for fattening (7-30 kg) (373 responses) | Yes |  | 4  (1.00) | 5  (0.63) | 2  (0.40) | 135  (0.96) | 14  (0.56) | 25  (0.15) | 3  (0.75) | 1  (0.25) | 0  (0.00) | 3  (0.21) | 192 |
|  |  | Directly | 1  (0.25) | 1  (0.20) | 0  (0.00) | 43  (0.32) | 7  (0.50) | 5  (0.20) | 1  (0.33) | 0  (0.00) | 0  (0.00) | 0  (0.00) | 58 |
|  |  | Delivery facilities | 2  (0.50) | 1  (0.20) | 2  (1.00) | 79  (0.59) | 7  (0.50) | 20  (0.80) | 1  (0.33) | 1  (1.00) | 0  (0.00) | 2  (1.00) | 115 |
|  |  | Delivery truck | 1  (0.25) | 3  (0.60) | 0  (0.00) | 11  (0.08) | 0  (0.00) | 0  (0.00) | 1  (0.33) | 0  (0.00) | 0  (0.00) | 0  (0.00) | 16 |
|  | No |  | 0  (0.00) | 3  (0.38) | 3  (0.60) | 6  (0.04) | 11  (0.44) | 138  (0.85) | 1  (0.25) | 3  (0.75) | 5  (1.00) | 11  (0.79) | 181 |
| Requirements for the truck on arrival | DANISH approved | Yes | 3  (0.75) | 4  (0.50) | 1  (0.20) | 69  (0.49) | 10  (0.40) | 21  (0.13) | 2  (0.50) | 1  (0.25) | 0  (0.00) | 1  (0.07) | 112 |
|  |  | No | 1  (0.25) | 4  (0.50) | 4  (0.80) | 72  (0.51) | 15  (0.60) | 142  (0.87) | 2  (0.50) | 3  (0.75) | 5  (1.00) | 13  (0.93) | 261 |
|  | Control of wash certificate | Yes | 2  (0.50) | 2  (0.25) | 2  (0.40) | 27  (0.19) | 5  (0.20) | 6  (0.04) | 0  (0.00) | 1  (0.25) | 0  (0.00) | 0  (0.00) | 45 |
|  |  | No | 2  (0.50) | 6  (0.75) | 3  (0.60) | 114  (0.81) | 20  (0.80) | 157  (0.96) | 4  (1.00) | 3  (0.75) | 5  (1.00) | 14  (1.00) | 328 |
|  | Clean and washed | Yes | 2  (0.50) | 3  (0.38) | 2  (0.40) | 65  (0.46) | 6  (0.24) | 15  (0.09) | 2  (0.50) | 1  (0.25) | 0  (0.00) | 1  (0.07) | 97 |
|  |  | No | 2  (0.50) | 5  (0.63) | 3  (0.60) | 76  (0.54) | 19  (0.76) | 148  (0.91) | 2  (0.50) | 3  (0.75) | 5  (1.00) | 13  (0.93) | 276 |
|  | First delivery of the day | Yes | 2  (0.50) | 3  (0.38) | 0  (0.00) | 31  (0.22) | 2  (0.08) | 6  (0.04) | 1  (0.25) | 1  (0.25) | 0  (0.00) | 0  (0.00) | 46 |
|  |  | No | 2  (0.50) | 5  (0.63) | 5  (1.00) | 110  (0.78) | 23  (0.92) | 157  (0.96) | 3  (0.75) | 3  (0.75) | 5  (1.00) | 14  (1.00) | 327 |
|  | No requirements (373 responses) |  | 0  (0.00) | 0  (0.00) | 0  (0.00) | 2  (0.01) | 0  (0.00) | 0  (0.00) | 1  (0.25) | 0  (0.00) | 0  (0.00) | 1  (0.07) | 4 |
|  |  |  | 4  (1.00) | 8  (1.00) | 5  (1.00) | 139  (0.99) | 25  (1.00) | 163  (1.00) | 3  (0.75) | 4  (1.00) | 5  (1.00) | 13  (0.93) | 369 |
| **Pigs for slaughter** |  |  |  |  |  |  |  |  |  |  |  |  |  |
| Farms delivering sows for slaughter | Yes |  | 4  (1.00) | 8  (1.00) | 1  (0.20) | 139  (1.00) | 24  (0.96) | 5  (0.03) | 4  (1.00) | 4  (1.00) | 0  (0.00) | 2  (0.14) | 191 |
|  |  | Directly | 0  (0.00) | 0  (0.00) | 0  (0.00) | 11  (0.08) | 1  (0.04) | 0  (0.00) | 0  (0.00) | 1  (0.25) | 0  (0.00) | 0  (0.00) | 13 |
|  |  | Delivery facilities | 1  (0.25) | 0  (0.00) | 0  (0.00) | 21  (0.15) | 4  (0.17) | 1  (0.20) | 0  (0.00) | 0  (0.00) | 0  (0.00) | 0  (0.00) | 27 |
|  |  | Delivery truck | 3  (0.75) | 8  (1.00) | 1  (1.00) | 107  (0.77) | 19  (0.79) | 4  (0.80) | 4  (1.00) | 3  (0.75) | 0  (0.00) | 2  (1.00) | 151 |
|  | No |  | 0  (0.00) | 0  (0.00) | 4  (0.80) | 0  (0.00) | 1  (0.04) | 158  (0.97) | 0  (0.00) | 0  (0.00) | 5  (1.00) | 12  (0.86) | 180 |
| How are sows transported for slaughter? | Owner transport |  | 0  (0.00) | 1  (0.13) | 0  (0.00) | 8  (0.06) | 1  (0.04) | 0  (0.00) | 0  (0.00) | 0  (0.00) | 0  (0.00) | 0  (0.00) | 10 |
|  | Slaughterhouse truck, often with pigs |  | 2  (0.50) | 3  (0.38) | 1  (1.00) | 91  (0.66) | 15  (0.63) | 5  (1.00) | 2  (0.50) | 2  (0.50) | 0  (0.00) | 2  (1.00) | 123 |
|  | Slaughterhouse truck, empty and washed |  | 0  (0.00) | 2  (0.25) | 0  (0.00) | 17  (0.12) | 3  (0.13) | 0  (0.00) | 2  (0.50 | 1  (0.25) | 0  (0.00) | 0  (0.00) | 25 |
|  | Slaughterhouse truck, empty, washed and first delivery of the day |  | 2  (0.50) | 2  (0.25) | 0  (0.00) | 22  (0.16) | 5  (0.21) | 0  (0.00) | 0  (0.00) | 1  (0.25) | 0  (0.00) | 0  (0.00) | 32 |
| Farms delivering finishers for slaughter | Yes |  | 1  (0.25) | 8  (1.00) | 4  (0.80) | 36  (0.35) | 24  (0.96) | 149  (0.91) | 2  (0.50) | 3  (0.75) | 5  (1.00) | 10  (0.71) | 242 |
|  |  | Directly | 0  (0.00) | 0  (0.00) | 0  (0.00) | 3  (0.08) | 2  (0.09) | 17  (0.11) | 0  (0.00) | 1  (0.33) | 1  (0.20) | 2  (0.20) | 26 |
|  |  | Delivery facilities | 0  (0.00) | 1  (0.13) | 1  (0.25) | 12  (0.33) | 18  (0.78) | 128  (0.86) | 0  (0.00) | 1  (0.33) | 3  (0.60) | 2  (0.20) | 166 |
|  |  | Delivery truck | 1  (1.00) | 7  (0.88) | 3  (0.75) | 21  (0.58) | 3  (0.13) | 4  (0.03) | 2  (1.00) | 1  (0.33) | 1  (0.20) | 6  (0.60) | 49 |
|  | No |  | 3  (0.75) | 0  (0.00) | 1  (0.20) | 103  (0.74) | 1  (0.04) | 14  (0.09) | 2  (0.50) | 1  (0.25) | 0  (0.00) | 4  (0.29) | 129 |
| How are finishers transported for slaughter? | Owner transport |  | 0  (0.00) | 0  (0.00) | 0  (0.00) | 1  (0.03) | 0  (0.00) | 6  (0.04) | 0  (0.00) | 0  (0.00) | 0  (0.00) | 5  (0.56 | 12 |
|  | Slaughterhouse truck, often with pigs |  | 1  (1.00) | 1  (0.13) | 1  (0.25) | 17  (0.49) | 3  (0.13) | 21  (0.14) | 1  (0.50) | 1  (0.33) | 1  (0.20) | 4  0.44 | 51 |
|  | Slaughterhouse truck, empty and washed |  | 0  (0.00) | 6  (0.75) | 3  (0.75) | 13  (0.37) | 17  (0.71) | 111  (0.74) | 1  (0.50) | 0  (0.00) | 3  (0.60) | 0  (0.00) | 154 |
|  | Slaughterhouse truck, empty, washed and first delivery of the day |  | 0  (0.00) | 1  (0.13) | 0  (0.00) | 4  (0.11) | 4  (0.17) | 11  (0.07) | 0  (0.00) | 2  (0.66) | 1  (0.20) | 0  (0.00) | 23 |
| Does the haulier enter the housing unit (beyond delivery room)? | Yes |  | 0  (0.00) | 0  (0.00) | 0  (0.00) | 0  (0.00) | 0  (0.00) | 4  (0.03) | 0  (0.00) | 0  (0.00) | 0  (0.00) | 1  (0.10) | 5 |
|  | No |  | 4  (1.00) | 8  (1.00) | 4  (1.00) | 139  (1.00) | 25  (1.00) | 146  (0.97) | 4  (1.00) | 4  (1.00) | 5  (1.00) | 9  (0.90) | 348 |
