# Supplementary material for: Indirect contacts between Danish pig farms – what are the frequencies and risk-reducing measures, and how can they be used in simulation models?
Source: Acta Vet Scand. 2025 Jan 24;67:7. doi: 10.1186/s13028-024-00789-z (PMC11762108; doi:10.1186/s13028-024-00789-z)
Supplement: Supplementary file 4 — Additional file 4. Questionnaire for veterinarians (translated from Danish) [file 13028_2024_789_MOESM4_ESM.docx]

**Additional file 4. Questionnaire for veterinarians** (translated from Danish)

Danish Agricultural & Food Council set a goal of increasing the number of PRRS-negative finishers and sow farms in Denmark. Therefore, SEGES is investigating potential risks of transmission. The questionnaire is targeted veterinarians specialized in pig practice, and includes:

- Planning of farm visits

- External biosecurity on-farm

- Handling and reaction on clinical signs of infectious diseases (e.g. PRRS, African swine fever and foot and mouth disease)

The responses will be used in the improvement of disease transmission models of the three diseases.

The questionnaire consists of 20 questions and lasts about 10-15 minutes to fulfill.

We thank you for your participation.

For further questions, please contact epidemiologist Mette Fertner, mefr@seges.dk

General data protection regulation:

Participation in the survey is voluntary, and you are free to omit reply of questions. Only projectpartners, employees in SEGES Innovation and University of Copenhagen, will have access to the responses. Personal data will not be passes on, and the data are deleted when they are no longer needed for the aim of the study.

***Organization of farm visits***

How many pig farms do you have Veterinary Health Advisory Service Contract with? __________

What percentage of the farms you visit are

- Sow farms (percent) __________
- Growers (percent) ___________
- Integrated (percent) __________

On how many of your visits to pig farms are you in direct physical contact with live/dead pigs (percentage)?

- 90-100 %
- 80-89 %
- 70-79 %
- 60-69 %
- 50-59 %
- 40-49 %
- 30-39 %
- 20-29 %
- 10-19 %
- 0-9 %

In which of the following regions do you practice? (multiple answers are possible)

- Northern Denmark Region
- Central Denmark Region
- Southern Denmark Region
- Region Zealand
- Capital Region of Denmark (including Bornholm)

What is the average distance (km) between two daily farm visits (different CHR numbers)?

Km (often) ____________

Km (max) _____________

How many daily farm visits (different CHR numbers) do you have?

- <1
- 2
- 3
- >3

On average, how many farm visits do you have per month? __________

External biosecurity

How many SPF farms have effective external biosecurity?

Percentage of the farms __________

How many conventional farms have external biosecurity in line with SPF requirements?

Percentage of the farms __________

How many registered quarantine sections meet the SPF requirements (in relation to, for example, air exchange, feed and slurry systems)?

Percentage of registered quarantine sections________

How many of the farms without a registered quarantine section still have effective quarantine procedures?

Percentage of the farms________

Managing and reacting to clinical signs of infectious diseases

What do you suspect are the three most likely routes of introduction of PRRS?

- Animal movements
- Wind
- Employees
- Sharing tools, e.g. washing robots
- Tradesmen
- Trucks and lorries
- Pest control
- Other

If signs of a clinical outbreak of PRRS are seen on a negative farm, how many farm owners would you expect to contact their veterinarian within

- the first two days (percentage of farmers)
- from day 2 to 7 (percentage of farmers)
- at the next scheduled farm visit (percentage of farmers)

Comments_______

If signs of a clinical outbreak of PRRS are seen on a negative farm, how many farmers do you estimate would wait to move their pigs within their own joint operation until the clinical suspicion has been confirmed/rejected?

Percentage of farmers____________

How many farmers do you expect would wait until clinical suspicion of PRRS has been confirmed/rejected before selling their pigs?

If cases of PRRS are seen on a negative farm, how many farmers do you estimate would wait until the clinical suspicion has been confirmed/rejected before selling pigs to other farms?

Percentage of farmers ____________

If Denmark had an outbreak of a notifiable pig disease such as foot-and-mouth disease or African swine fever, how many farmers do you suspect would follow the restrictions on animal movements (i.e. that animals are only moved if an exemption has been granted by The Danish Veterinary and Food Administration)?

- From when the restrictions are announced and two days ahead? (percentage of farmers) _______
- After two days and up to one week after? (percentage of farmers) _______
- After one week? (percentage of farmers) _______

Do you work with other species than pigs?

- Yes
- No

How often are you called in for emergency visits to pig farms?

- Daily
- Weekly
- Monthly
- Yearly
- Never
- Not relevant

How much of the last six months have you spent working with the following species?

- Pigs (percentage)
- Cattle (percentage)
- Small ruminants (percentage)
- Poultry (percentage)
- Family pets (percentage)
- Other (percentage)

How often is your visit to pig farms followed by a visit to one of the following farm types (the same day)?

- Pig farm (percentage)
- Cattle farm (percentage)
- Poultry farm (percentage)
- Small ruminant farm (percentage)

How often is your visit to cattle farms followed by a visit to one of the following farm types (the same day)?

- Pig farm (percentage) ___________
- Cattle farm (percentage) __________
- Poultry farm (percentage) __________
- Small ruminant farm (percentage) __________

How often are you called for emergency visits to the following farm types?

- Pig farms (Daily, Weekly, Monthly, Yearly, Never, Not relevant)
- Cattle farms (Daily, Weekly, Monthly, Yearly, Never, Not relevant)
- Poultry farms (Daily, Weekly, Monthly, Yearly, Never, Not relevant)
- Small ruminant farms (Daily, Weekly, Monthly, Yearly, Never, Not relevant)
