# Supplementary material for: Indirect contacts between Danish pig farms – what are the frequencies and risk-reducing measures, and how can they be used in simulation models?
Source: Acta Vet Scand. 2025 Jan 24;67:7. doi: 10.1186/s13028-024-00789-z (PMC11762108; doi:10.1186/s13028-024-00789-z)
Supplement: Supplementary file 5 — Additional file 5. Questionnaire for pig farmers (translated from Danish) [file 13028_2024_789_MOESM5_ESM.docx]

**Additional file 5. Questionnaire for pig farmers** (translated from Danish)

SEGES Innovation is investigating transmission pathways for PRRS. The questionnaire deals with employees, visitors, equipment and movements of pigs to and from the CHR-number.

The questionnaire consists of 25 questions and takes approximately 10 minutes to complete. We thank you for your participation.

If you have any questions, you are very welcome to contact epidemiologist Mette Fertner, mefr@seges.dk.

General data protection regulation:

Participation in the survey is voluntary, and you are free to omit reply of questions. Only projectpartners, employees in SEGES Innovation and University of Copenhagen, will have access to the responses. Personal data will not be passes on, and the data are deleted when they are no longer needed for the aim of the study.

You have to respond the questionnaire for a single CHR-number you are working on or own. If you have more than one CHR-number, you are more than welcome to fill out a questionnaire for each CHR-number. It is only possible to complete one questionnaire per CHR-number.

All filled questionnaires participate in the competition of a gift voucher by a lottery.

CHR number (only one answer per CHR number) _________

How many visitors enter the housing unit during a typical month (excluding the veterinarian), for example consultants, tradesmen and other visitors? Specify the number of visitors per month __________

Do the employees work on other farms (with other CHR numbers)?

- Yes
- Yes, but only on farms that are part of the same joint operation
- No

How many farms are covered by shared employees? __________

How far apart are the farms located? ___________

Do dogs/cats enter the farm, and can they enter/exit the housing unit?

- Yes
- No
- Don’t know

Do birds enter the housing unit?

- Yes
- No
- Don’t know

Does the farm share equipment or trucks (within the farm) with other CHR numbers? (e.g. washing robots, skid steer loaders)

- Yes
- Yes, but only with farms that are part of the same joint operation
- No

How many farms share equipment or trucks? ___________

How far apart are farms that share tools/trucks? (specify in km) __________

Are pigs purchased for the farm?

- Yes, 7 kg
- Yes, 30 kg
- Yes, gilts
- No

Are gilts placed in a quarantine unit on arrival?

- Yes
- Most of them
- No

How long do the gilts remain in the quarantine unit? ____________

Who is normally responsible for the transport of the pigs during purchase?

- Private transport (buyer or seller)
- SPF
- DANISH approved
- Other pig transporting company
- Other ___________

Does the farm deliver pigs for fattening? (7 kg or 30 kg pigs)

- Yes
- No

When delivering pigs for fattening, are there any requirements for the vehicles on arrival at the farm? (Multiple answers possible)

- Use only DANISH certified transporters
- check washing certificate
- Yes, empty and washed clean
- Yes, first load of the day
- No
- Other
- Comments

How are pigs for fattening delivered?

- Direct delivery with the possibility of the pigs running back to the housing unit
- Via delivery facilities on the farm (e.g. delivery room, loading ramp)
- Delivery truck
- Other __________

Does the farm deliver sows for slaughter?

- Yes
- No

How are sows for slaughter delivered?

- Direct delivery with the possibility of the pigs running back to the housing unit
- Via delivery facilities on the farm (e.g. delivery room, loading ramp)
- Delivery truck
- Other __________

How are the sows transported to the slaughterhouse?

- Owner transport
- Slaughterhouse truck, often with pigs
- Slaughterhouse truck, empty and washed
- Slaughterhouse truck, empty, washed and first delivery of the day
- Other _________

Does the farm deliver pigs for slaughter?

- Yes
- No

How are pigs for slaughter delivered?

- Owner transport
- Slaughterhouse truck, often with pigs
- Slaughterhouse truck, empty and washed
- Slaughterhouse truck, empty, washed and first delivery of the day
- Other __________

Does the haulier enter the housing unit (besides the delivery room) when loading the pigs?

- Yes
- No

How far from the farm are carcasses collected?

- Less than 50 metres
- More than 50 metres

How often is feed delivered to the farm?

- Daily
- Weekly
- Monthly
- Other _________

Does the farm deliver manure for biogas?

- Yes
- No
- Don’t know

How many times a month does the farm deliver manure for biogas? ­­­­­­­________
